# The effect of government information release on public protective behaviors during the emerging infectious diseases epidemic in China

**DOI:** 10.3389/fpubh.2026.1731670

**Published:** 2026-03-25

**Authors:** Yi Chun Li, Pan Tang

**Affiliations:** 1 School of Public Administration and Emergency Management, Jinan University, Guangzhou, China; 2 School of International Relations and Public Affairs, Fudan University, Shanghai, China

**Keywords:** compliance behaviors, epidemic prevention and control, information release, protective behaviors, risk communication

## Abstract

**Background:**

Cultivating public protective behaviors is essential for the successful prevention and control of emerging infectious diseases epidemics, such as COVID-19. Government information release plays an important role in promoting proactive public protective behaviors. The existing literature lacks comprehensive understanding of the impact of government information release on public protective behaviors under different conditions.

**Methods:**

This research proposes a number of research hypotheses for the effect of government information release on public protective behaviors mediated by risk perception and institutional trust. The Mediator, also known as the mediating variable, is a significant statistical concept employed to analyze the mechanism of influence between the independent and dependent variables.

**Results:**

The empirical research during the normalization phase of COVID-19 epidemics prevention and control in China indicates that government information release significantly influences public protective behaviors by the mediating roles of risk perception and institutional trust.

**Conclusion:**

The research provides insights for designing strategies for cultivating public protective behaviors from the perspective from the information release and fostering institutional trust.

## 1 Introduction

Emerging infectious diseases present significant challenge to global public health in the 21st century, affecting the whole society of each country worldwide (1). Contributing factors such as climate change, environmental pollution, globalization, and urbanization have increased the risk of epidemic outbreaks and pandemics (2). Unlike conventional emergencies, public health crises, particularly those caused by infectious diseases, introduce unique complexities that present significant governance challenges. One key difficulty lies in the complex interaction between causative factors and affected populations, which complicates epidemic control efforts. The public not only serves as carriers of the virus but also as vulnerable populations. Research has shown that proactive protective behaviors, such as hand hygiene and social distancing, can significantly reduce the risk of virus transmission (3). As a result, adherence to recommended preventive measures, including mask-wearing and social distancing, is crucial to halting the spread of infectious diseases (3–5).

However, public risk perception often lags behind objective changes, heavily relying on information from governmental authorities. Given the hidden nature of virus transmission (6) and the incubation period, risk assessment is often delayed. Thus, proactive government information release and effective risk communication are essential for controlling infectious diseases (7). In China, the government plays a central role in providing specialized information on epidemic prevalence and viral mechanisms, which influences public perception and guides prevention efforts.

**Figure 1:**
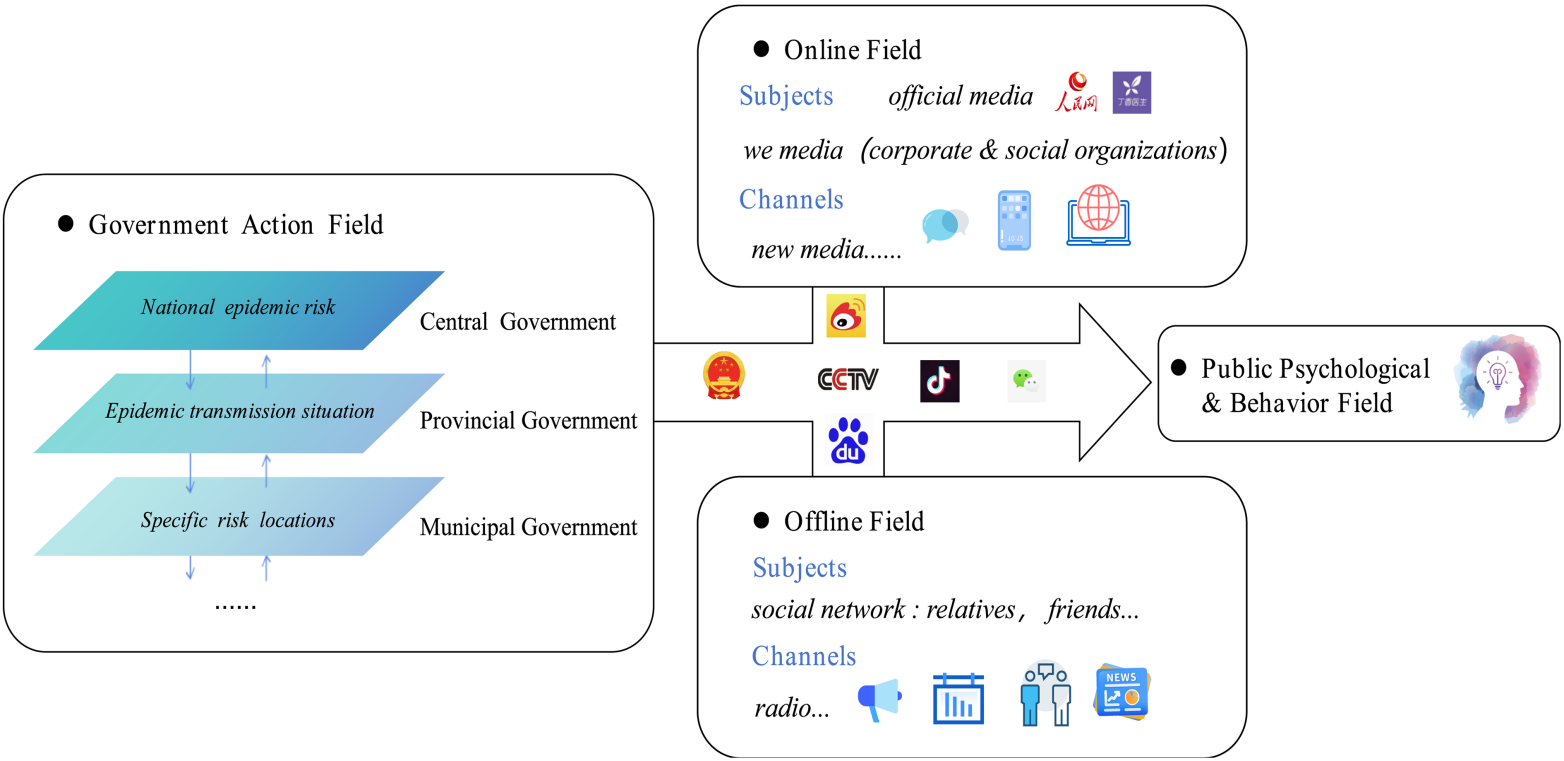
Information communication network during the COVID-19 pandemic.

Non-mandatory risk communication measures, such as information release, are practical, cost-effective, adaptable, and easy to implement (8–10). These measures serve as a key means for the public to understand governmental actions (11, 12–14). During the COVID-19 pandemic, governments issued messages to prompt action and encourage protective behaviors such as mask-wearing and social distancing to reduce the spread of the virus (15). In China, a public health emergency alert and notification system has been established. To ensure effective communication, the government, media, and society have created a comprehensive information network that incorporates both traditional and new media channels (see Figure 1). At various levels, the government takes the lead in disseminating information, while the media and society act as intermediaries, and the public serves as the recipient. Information is distributed through platforms like micro-blogs, WeChat, government websites, television broadcasts, and community updates. Despite the diversity of communication methods, discrepancies in public protective behaviors remain. These discrepancies reflect variations in behavioral responses, leading to gaps between actual behaviors and established standards, and even contributing to negative behaviors that facilitate virus transmission.

Understanding the diversity and complexity of public behavior, and uncovering the underlying mechanisms, are essential theoretical endeavors. While research has extensively explored the factors influencing public protective behaviors through cognitive psychology, there is a relative lack of empirical studies that incorporate government information release and other risk communication measures as explanatory variables. Additionally, many existing studies fail to consider social environmental factors, which complicates explanations based solely on psychological theories. This led to contradictory findings, such as negative or insignificant effects of risk perception, government trust, and policy interventions on public behavior, difficult to explain the complexity of the impact of variables such as information sources (16–20, 21). A more systematic analysis of the mechanisms behind public protective behaviors—considering risk, social environments, and the efficacy of government risk communication—is crucial for accurately understanding variations in public behavior. Only by employing such an approach can we effectively clarify the factors influencing these behaviors.

Facing to the theoretical and practical challenges, this research focuses on the normalization stage of COVID-19 in China as a case study. Since May 2020, China has entered the normalization stage of epidemic prevention and control, marked by sporadic outbreaks and occasional clusters caused by localized cases, effective control of imported infections, and the sustained consolidation of positive trends in epidemic management. The data for this study was collected from September 2022 to October 2022, a period during which notable discrepancies in risk zoning across public areas were observed. During this time, most regions were classified as low-risk, with public spaces fully open and daily life largely returning to normal. However, these areas still faced the potential threat of imported COVID-19 cases. In contrast, some regions were designated as moderate to high-risk areas, where the risk of transmission remained elevated. This timeframe provided a valuable opportunity to examine the diversity and complexity of public behavior across different risk zones.

The primary objectives of this study are to explore the factors influencing public protective behaviors, formulate research hypotheses, construct a mediation model, and investigate the mechanisms through which information dissemination impacts public protective actions. Furthermore, based on the findings, this study aims to offer recommendations for optimizing policy guidance and contribute theoretical insights into establishing a collaborative emergency governance framework between the government and the public.

## 2 Literature review

### 2.1 The conceptual connotation of public protective behavior

In the research on emergency management, public protective behavior is a type of risk response behavior, representing the efforts made by individuals in the face of a risk situation. This behavior primarily aims to reduce risk, encompasses both short-term disaster response (e.g., emergency evacuation and retreat from hazardous areas) and long-term preparedness actions (stock emergency supplies and purchase disaster insurance), may manifest as active engagement, or as passive avoidance and inaction (22, 14, 23).

Public protective behavior is complex, based on an intricate cognitive and psychological process, and influenced by multiple factors. While earlier research often relied on the “rational man” hypothesis, assuming purely logical risk decision-making, the actual process is much more complex, involving personal preferences, background knowledge, and cognitive limitations (24). Recent psychological models on public behavior (refer to Table 1) highlight the mediating effects of cognitive processes.

**Table 1:** Psychological theories of disaster behavior decision mechanisms.

Important theory	Decisive factors of behavior
Expectancy theory	Value and likelihood of the outcome (90).
Theory of reasoned action	Behavioral motivation/intention, influenced by attitudes and norms (91).
Protection motivation theory	Two cognitive processes: Threat appraisal & Coping appraisal (92).
Protective action decision model	Social background, environmental factors, information, and experiences (22).

Focus on the field of public health, protective behavior refers to actions taken by individuals to prevent the spread and progression of diseases (25–28). Facing the spread and diffusion of infectious diseases, public managers can effectively influence and shape the public’s protective behaviors through effective risk communication measures (29–32). Risk communication involves the exchange of information among various stakeholders—government, the public, scientists, media, and social organizations—regarding the nature, severity, significance, or management of risks (10, 33). This encompasses not only direct risk information but also opinions expressed on emergencies or government regulations and measures. The government can effectively convey crisis-related information through clear explanations, narrative techniques, and proposed solutions, helping the public understand and adapt to crises. This, in turn, enhances the persuasiveness of policies, builds public trust, and guides protective behavior (29–31). To explore the impact mechanism of risk communication measures on the protective behaviors, this study defines the protective behaviors of the Chinese public during the COVID-19 pandemic as: the compliance behaviors of individuals in response to the risk of COVID-19, aimed at mitigating the impact of the risk on themselves in accordance with the government’s protective policy standards, including practices such as social distancing, adhering to protective measures when going out, and maintaining proper hygiene (34, 35).

From this perspective, public behavior can be categorized into two patterns: risk coping and institutional compliance. Risk Coping involves individuals’ efforts to mitigate risks when confronted with hazardous situations (36), including actions to reduce vulnerability (e.g., wearing masks) and behaviors to build resilience (e.g., maintaining a healthy lifestyle) (22).

Compliance refers to individuals aligning their behaviors with prescribed guidelines, characterized by proactive, internal motivations (37, 38). In disaster situations, higher trust in government often leads to increased willingness to engage in preparatory actions and to rate the government’s crisis response more favorably (37). Those with greater trust in the government tend to be more proactive in mitigating risks (39, 40, 41).

### 2.2 Influencing factors of public protective behavior

Empirical research has extensively examined the factors influencing public protective behavior during the COVID-19 pandemic, focusing primarily on four aspects: risk perception, information communication, government trust, and individual characteristics.

*Risk perception*: the perceived severity of infection plays a crucial role in influencing protective behaviors (42, 43). A higher risk perception is generally associated with greater adoption of protective behaviors (3, 9, 25, 44, 45). However, some studies observed that during the second wave of the pandemic, an increased risk perception did not always translate into protective behaviors (19, 46), and negative economic risk perception was sometimes inversely related to protective actions (18).

*Information communication*: risk communication and the dissemination of risk-related information significantly influence public cognition and, consequently, behavior. Information from the government and media alters risk perception and encourages preparedness (28). Different media channels have varying effects on public cognition and behavior (47). For instance, exposure to mass media during the COVID-19 pandemic was associated with increased adherence to hand hygiene practices (48). Research demonstrates that accessing COVID-19 information through online resources promotes preventive behaviors (21), with further evidence showing that new media and interpersonal communication are more effective than traditional media in fostering vaccination intentions via health beliefs (47),

*Government trust*: public trust in government institutions plays a key role in fostering protective behavior, given the government’s central role in managing public health emergencies (49). However, excessive trust in authorities may have a counterproductive effect, diminishing individual preparedness for disaster mitigation (50–52). Furthermore, some studies indicate that government trust could reduce risk perception, potentially undermining public protective behaviors (12, 16).

*Individual characteristics*: personal factors such as gender, residence, age, education level, disaster experience, and knowledge significantly influence protective behaviors (26, 48, 53–55). For example, individuals with higher education levels are more likely to engage in proactive protective behaviors (26).

Despite the considerable progress made in identifying the variables that influence public protective behaviors during the COVID-19 pandemic, several gaps remain. First, while the impact of government information release and risk communication on public behavior is well-documented (7, 56), empirical research specifically focused on government risk communication, particularly information release as a core variable, is still limited. There is a notable lack of exploration into its mechanisms and effects on public behavior. Second, many studies have primarily relied on cognitive psychology theories to analyze the influencing mechanisms behind public behavior, often overlooking broader systemic factors, such as social context and interpersonal dynamics (e.g., conformity effects). This narrow focus has led to conflicting conclusions and discrepancies, particularly regarding the influence of risk perception and government trust on public behavior.

Building on these observations, this research aims to comprehensively examine the factors influencing public protective behaviors, with a particular focus on government information release as a critical research variable. By exploring these factors, the study seeks to provide deeper insights into the diversity of public protective behaviors and the underlying mechanisms that drive them.

## 3 Hypothesis

Protective behavior refers to voluntary actions taken by the public to mitigate the risk of contracting the novel coronavirus, reflecting responses to pandemic risks and regulatory guidelines (22). This study focuses on government risk communication measures, explores the relationship between government information release and public protective behavior. From a cognitive psychology perspective, it posits that government risk communication affects public behavior primarily by influencing psychological cognition, influenced by different channels and agents in risk communication.

### 3.1 Information release quality and protective behavior

Previous literature highlights the significant impact of government risk communication activities on public disaster response and risk mitigation (12–14). During the COVID-19 pandemic, government-released information, such as epidemic updates and disease control measures, was crucial for shaping public behavior. According to the explanatory level theory, public attitudes and behaviors towards government policies depend on the level of interpretation of the policy content. A higher level of interpretation improves the public’s understanding and awareness of government actions, leading to greater support and compliance. Conversely, a lower level of interpretation may foster non-compliant behaviors (57, 58). Government-released information should be comprehensive, timely, and effective (59–61). The quality of government information—including its utility, objectivity, and completeness—depends on factors such as scope, content, timing, and release methods (62). Low-quality or ineffective risk information can create uncertainty, negatively impacting public protective behaviors, such as adherence to social distancing guidelines (63). Thus, public perception of information release quality, including its timeliness, responsiveness, and alignment with public needs, significantly influences protective behavior. Based on these observations, this study posits the following hypothesis:

*H1*: The public’s perception of the quality of government information release (Information Release Quality) positively influences protective behavior.

### 3.2 The mediating role of cognitive processes: risk perception and institutional trust

Risk information is fundamental to public perception and decision-making processes (22). Many factors, especially risk communication, influence public risk perception (7, 63, 64). The perceived quality of government information release directly affects how the public perceives the epidemic’s risk. Higher perceived risk is associated with increased compliance with risk-mitigating behaviors. Based on this, we propose the following hypotheses:

*H2.1*: The perceived quality of government information release positively influences epidemic risk perception.

*H2.2*: Risk perception positively influences protective behaviors.

*H2.3*: Risk perception mediates the relationship between perceived information release quality and protective behaviors.

Trust in government and institutions is essential for encouraging compliance with protective behaviors. High levels of institutional trust enhance the public’s belief in the effectiveness of government measures, promoting proactive behaviors (12, 37, 65). Effective communication helps build trust, which influences adaptive behavior (40). This study posits the following hypotheses regarding institutional trust:

*H3.1*: The perceived quality of government information release positively influences trust in epidemic prevention measures.

*H3.2*: Trust in epidemic prevention measures positively influences protective behavior.

*H3.3*: Trust in epidemic prevention measures mediates the relationship between perceived quality of information release and protective behavior.

### 3.3 Communication process: information channels and agents

The choice of communication channel significantly impacts public cognition and behavior. Different media (traditional vs. new) and communication agents (experts vs. government) influence public awareness and adherence to prevention measures (66–69). During the COVID-19 pandemic, social media emerged as a pivotal tool for rapid communication and information dissemination (33, 70), leveraging the advantages of social media platforms, the government can achieve rapid transmission of accurate information (71). Moreover, risk information can be sourced from diverse agents, including government agencies, experts, healthcare professionals, or social networks (72–74). Across various countries, individuals commonly place trust in doctors or medical experts for information related to infectious diseases (75, 76). Additionally, research indicates that government social media accounts play a pivotal role in fostering compliant behavior, promoting adherence to public policies, and encouraging self-protection during public health crises (8). Hence, communication agents with greater authority are more likely to garner public trust, thereby guiding compliance behavior: The following hypotheses address the role of information channels and agents:

*H4.1*: Compared to traditional media (e.g., television, radio), the level of protective behavior is higher among individuals who acquire epidemic information through mobile applications.

*H4.2*: Public engagement with authoritative communication agents (e.g., experts, government officials, and official media) leads to higher levels of protective behavior.

### 3.4 Control variables and alternative hypotheses

This study controls for several variables that could influence public behavior, such as regional differences, pandemic risk levels, herd and social conformity effects, and individual characteristics (e.g., age, gender, education, risk preferences, and disaster experiences) (26, 53, 54, 48, 55, 77). The study will also account for the potential environmental and individual trait variables that may impact public protective behavior.

**Figure 2:**
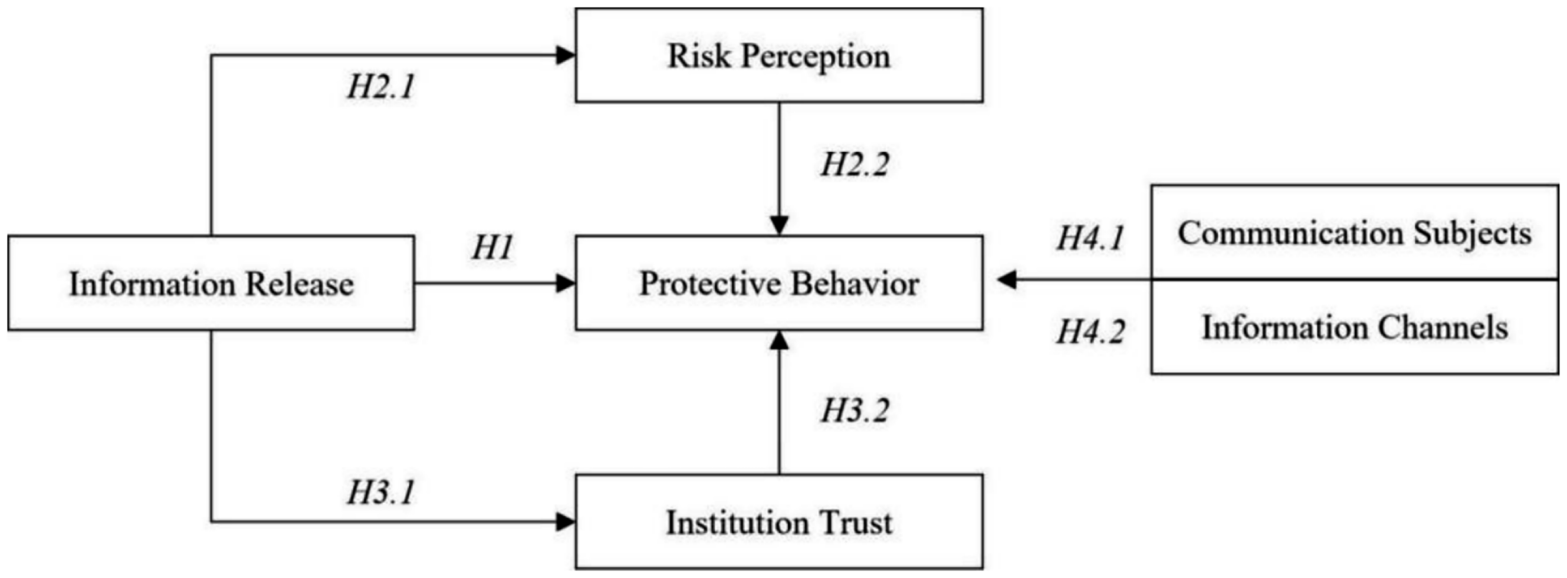
The research hypothesis.

In conclusion, the research framework of this study is illustrated in Figure 2.

## 4 Research design

### 4.1 Variables and measures

In this research, the dependent variable is public protective behavior. It refers to the self-protective actions individuals undertake in response to the risk of contracting COVID-19. This study has identified 5 representative behavioral criteria to observe the public’s protective behavior, aiming to assess the consistency between the public’s actions and policy standards.

The independent variables are the characteristics of information release. First, the information release quality refers to the subjective assessment by the public, as information recipients, regarding the timeliness, responsiveness, and effectiveness of government information release activities. Timeliness evaluates the speed of information dissemination, responsiveness assesses the government’s level of addressing public concerns, and effectiveness examines whether the information meets the public’s needs.

The mediating variables are institution trust and risk perception. First, the institution trust refers to the subjective perception and evaluation of the public regarding the capability and effectiveness of epidemic prevention measures. This encompasses three dimensions, such as capability trust, which evaluates whether policies have achieved epidemic prevention and control objectives and to what extent; public interest trust, which assesses whether epidemic prevention and control policies have served the public interest, the degree of serving public interest, or causing harm to it; and executing entity trust, which evaluates the effectiveness of government staff’s accountability. Second, this paper assesses the level of Risk Perception based on the public’s evaluation of the degree of concern, severity, and impact of the epidemic. The measurement of these variables adopts a 7-point likert scale, where 1 = strongly disagree and 7 = strongly agree, with the average value of the questions recorded as the variable’s final value.

The communication subjects are the important sources through which the public acquires information, primarily including experts, self-media, government, official media, and offline social network. The information channels mainly consist of mobile applications, television, radio, internet portals, and other mediums.

This research also incorporates risk preference, disaster experience, others’ coping behavior, as well as gender, age, education, and region as control variables. The measurement of all the aforementioned research variables are presented in Table 2.

**Table 2:** Measurement of research variables.

Variables	Operationalization	Items
Protective behavior	Mask-wearing during outings	You always wear a mask when going out.
	Cleaning and disinfection	You disinfect after receiving deliveries and wash your hands afterward.
	Maintaining a safe distance	You try to maintain a safe distance from others while queuing.
	Attempting to avoid gatherings	You try to avoid attending gatherings as much as possible.
	Maintaining a healthy lifestyle	During the epidemic, you improve your immunity through nutritional supplements and physical exercise.
Information release quality	Speed of information release	You believe that the government promptly discloses confirmed cases and investigation information.
	Timely response to inquiries	You believe that the government quickly responds to epidemic-related concerns from the public.
	Meeting information demands	Government’s release of epidemic information enables you to fully understand the main developments of the epidemic.
Risk perception	Level of concern on epidemic	You are worried about contracting the coronavirus.
	Severity of the epidemic	You consider the COVID-19 to be very serious.
	Impact of the epidemic	You believe that being infected would significantly affect your health or reputation.
Institution trust	Trust in public interest	You believe that the government’s epidemic prevention and control measures are to protect you from being infected.
	Trust in institutional capability	You believe that the government’s prevention and control measures can effectively control the spread of the epidemic.
	Trust in executive bodies	You believe that government officials have been actively fulfilling their duties in epidemic prevention.
Communication subjects	Perception of communicators	Experts, Self-media, Government, Official media, Family and friends; Others
Information channels	Perception of channels	Mobile apps, Television, Radio, Internet portals, Others
Risk preference	Degree of Risk Aversion	After noticing 10 new asymptomatic infections in your city, please rate the epidemic risk on a scale of ___ (0–10)
Disaster experience	Experience of epidemic	Have you ever been quarantined as a close or secondary close contact?
Regional risk level	Epidemic control measures	Is there a restricted area due to the epidemic in your district or county?
Regional fixed effects	Province fixed effects	Your province of residence?
Others’ coping behaviors	Compliance of others	How well do the people around you adhere to the behaviors?
Demographic variables	Gender, age, education	Your gender, your age, your level of education?

### 4.2 Data source

This research employed a combined online and offline approach for data collection, using a questionnaire survey conducted in July and August 2020. The online survey was distributed via “Wen Juan Wang,” one of China’s largest online survey platforms. This platform offers a diverse sample database that includes respondents from various cities, regions, income levels, ages, and occupations across the country, ensuring a broad and representative respondent pool. The platform’s comprehensive sample database helped guarantee the authenticity and quality of the collected data.

**Figure 3:**
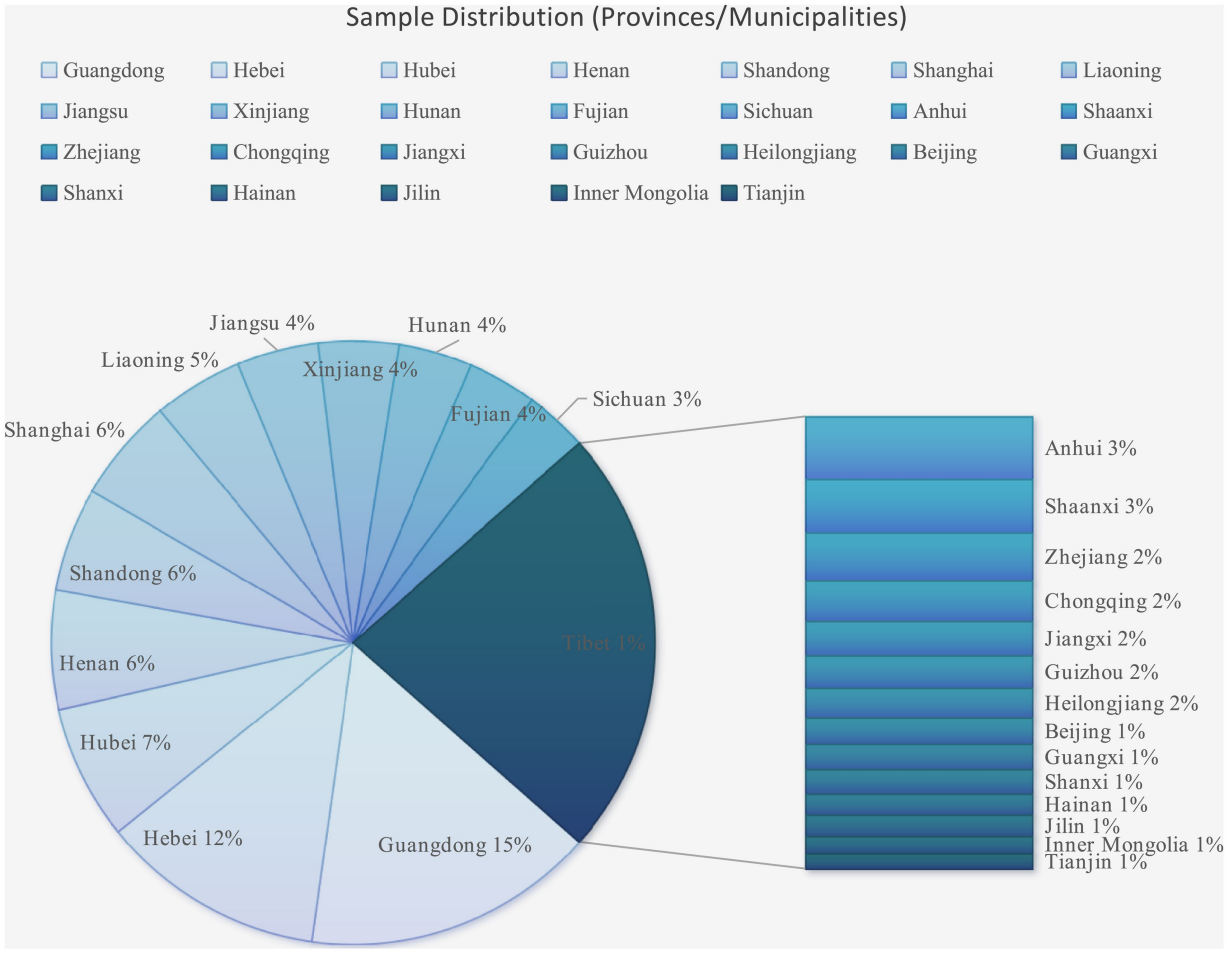
Sample distribution.

A total of 1,140 responses were gathered, with 1,054 online responses and 76 offline paper-based responses. After eliminating incomplete or invalid samples, 926 valid responses remained, resulting in an effective response rate of 81%. The geographical distribution of the sample is presented in Figure 3, with further details provided in Table 3.

**Table 3:** The sociodemographic characteristics of respondents.

Variable	Frequency	Percentage (%)
Gender		
Male	379	40.93
Female	547	59.07
Age (year groups)		
≤17	17	1.84
18 ~ 25	358	38.66
26 ~ 40	475	51.30
41 ~ 60	74	7.99
≥61	2	0.22
Education (level groups)		
Elementary school and below	8	0.86
Junior high school	30	3.24
High/vocational school	108	11.66
Associate degree	220	23.76
Bachelor’s degree and above	560	60.47
Total	926	100.00

In the survey, the majority of respondents (69.65%) primarily rely on mobile applications, such as Weibo, WeChat, and various news apps, to access epidemic-related information. Other commonly used sources include internet portal websites (54.97%), television (48.38%), and radio (29.05%). When it comes to the subjects or types of information sources, self-media platforms were the most familiar to the public, with 76.24% of respondents indicating familiarity. This was followed by official media (69.76%), government sources (65.23%), experts (34.45%), and friends or relatives (19.65%), as shown in Table 4.

**Table 4:** Channels and subjects.

Variable	Frequency	Proportion (*n* = 926)
Information channels		
Mobile apps	645	69.65%
Television	448	48.38%
Radio	269	29.05%
Internet portals	509	54.97%
Other	24	2.59%
Communication subjects		
Experts	319	34.45%
Self-media	706	76.24%
Government	604	65.23%
Official media	646	69.76%
Family and friends	182	19.65%
Other	22	2.38%

### 4.3 Research method

**Figure 4:**
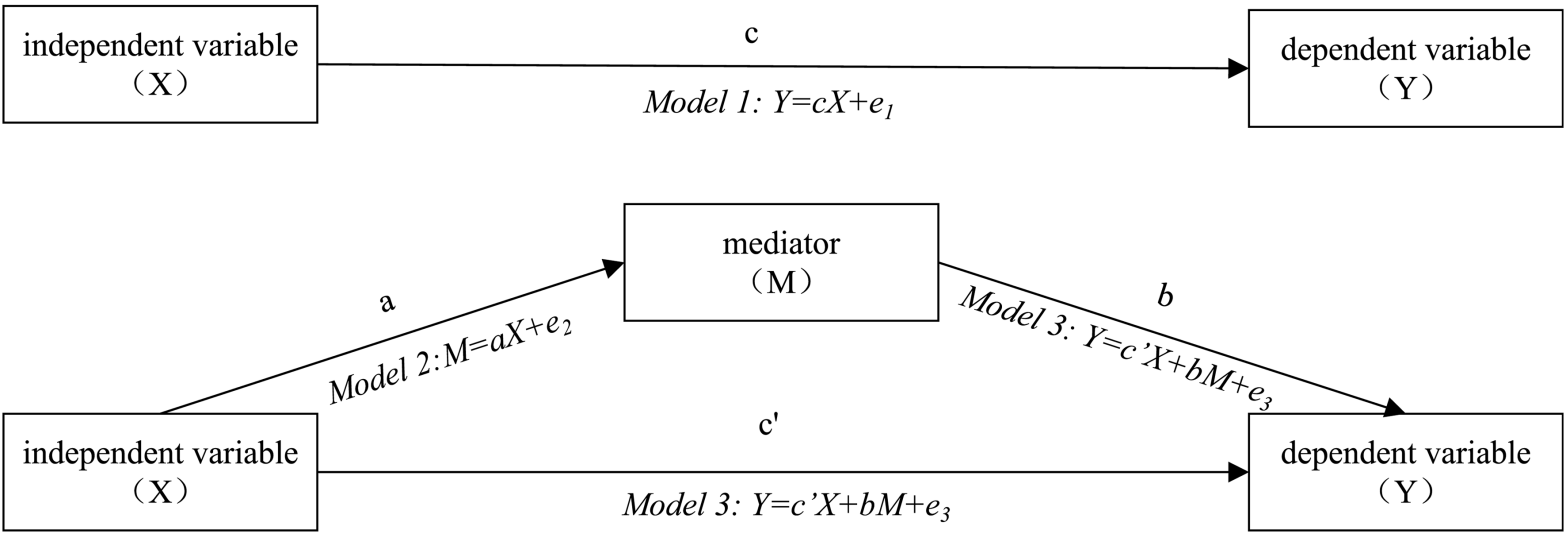
The mediation effects model.

The Mediator, also known as the mediating variable, is a significant statistical concept employed to analyze the mechanism of influence between the independent and dependent variables. The primary model is illustrated in Figure 4. In this model, “c” represents the total effect of X on Y, which is the coefficient of the effect of the independent variable on the dependent variable without considering the intermediary variable. ‘a*b’ represents the mediating effect, while ‘c” stands for the direct effect of X on Y, which is the coefficient of the effect of the independent variable on the dependent variable considering the intermediary variable (78).

Regarding the analysis of the mediation effect, two common methods are utilized: the causal step regression test and the product coefficient test. The causal step regression method is widely employed due to its operational simplicity. However, it is contentious as it is prone to Type I errors and cannot provide confidence intervals for the mediation effect (79, 80, 81). On the other hand, the product coefficient test assesses the significance of ‘a*b’, which includes the Sobel test and the Bootstrap method. The Sobel test has stringent requirements, often challenging to meet in practice (such as the assumption of normal distribution for the original data and ‘a*b’ data, and the necessity of a large sample size), thereby diminishing accuracy (82, 81, 83). In contrast, the Bootstrap method imposes no restrictions on the sampling distribution. It offers more precise confidence intervals by resampling the observed data, calculating the estimator’s variance, constructing its confidence interval, and then making statistical inferences about population characteristics. This method examines whether the 95% confidence interval of the regression coefficient ‘a*b’ includes the number 0. If the interval excludes 0, it suggests a mediation effect; conversely, if the interval includes 0, it indicates no mediation effect (80, 84).

**Figure 5:**
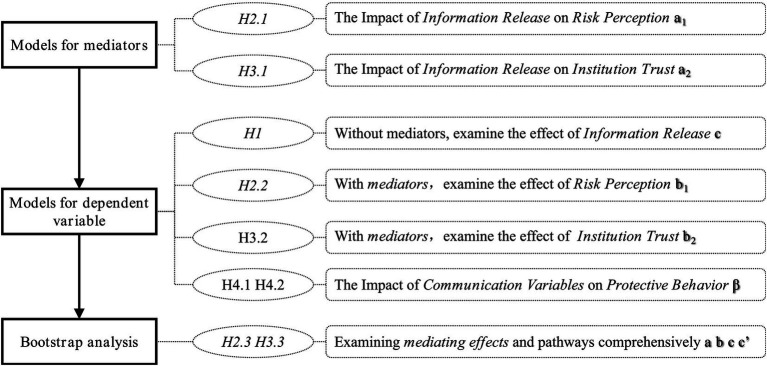
The procedure for testing hypothesis.

To provide a coherent hypothesis testing strategy, this paper adopts an approach proposed by scholars Wen and Ye (85), integrating the causal step regression test with the Bootstrap method, following the steps outlined in Figure 5: Step 1 involves constructing a regression model for the independent variable and the mediators to test H2.1 and H3.1. Step 2 entails separately constructing regression models for the independent variable and the dependent variable, and for the independent variable, mediators, and dependent variable to test H1, H2.2, and H3.2 (while concurrently examining the information communication process H4.1, H4.2 based on the regression model of the dependent variable). Step 3 involves utilizing the Bootstrap method to comprehensively analyze the impact of the intermediary effect.

This research conducted reliability and validity analyses for the scales of the independent variable, mediator variables, and dependent variable, as shown in Table 3. The reliability coefficients indicate that the Cronbach’s alpha values for all variables exceed the critical threshold of 0.7. The results of the factor analysis reveal a Kaiser–Meyer–Olkin (KMO) value of 0.931, significant at *p* < 0.01, with a cumulative variance explained of 66.22%. Furthermore, the consistency between individual items and corresponding factors aligns with the theoretical framework, indicating good construct validity of the questionnaire scale (Table 5).

**Table 5:** Reliability and validity analysis.

Variables	Items	Factor loadings				Cronbach’s alpha	Validity coefficient
		F1^1^	F2	F3	F4		
Protective behavior	Item1 mask-wearing during outings	0.591				0.823	KMO = 0.931 cumulative variance 66.22% *p* < 0.001
	Item2 cleaning and disinfection	0.805					
	Item3 maintaining a safe distance	0.687					
	Item4 attempting to avoid gatherings	0.592					
	Item5 maintaining a healthy lifestyle	0.554					
Information release quality	Item1 speed of information release		0.805			0.813	
	Item2 timely response to inquiries		0.742				
	Item3 meeting information demands		0.705				
Risk perception	Item1 level of concern on epidemic			0.740		0.784	
	Item2 severity of the epidemic			0.710			
	Item3 impact of the epidemic			0.686			
Institution trust	Item1 trust in public interest				0.798	0.701	
	Item2 trust in institutional capability				0.602		
	Item3 trust in executive bodies				0.790		

1 FI: Factor 1.

## 5 Analysis results

### 5.1 Descriptive statistics

The descriptive analysis of the independent, mediating, and dependent variables (see Table 6) reveals significant and strong correlations, with Pearson correlation coefficients exceeding 0.5, among Information Release Quality, Risk Perception, Institution Trust, and Protective Behavior. Additionally, strong correlations were also observed between Risk Perception, Institution Trust, and Protective Behavior, further supporting the proposed research hypotheses.

**Table 6:** Inter-correlations among the key variables.

Variable	M	SD	1	2	3	4
1. Protective behavior	5.887	0.841	1			
2. Risk perception	5.518	1.019	0.512^**^	1		
3. Institution trust	5.767	0.885	0.761^**^	0.491^**^	1	
4. Information release quality	5.743	0.903	0.722^**^	0.468^**^	0.739^**^	1

*p < 0.05, **p < 0.01, two-tailed.

### 5.2 Regression analysis

The regression models were employed to examine the relationship between information release quality, risk perception, and institutional trust, as well as the relationship between information release quality and risk perception (Model 1) as well as institution trust (Model 2) (see Table 7). The results show that information release quality influences both risk perception and institution trust significantly and positively. The research hypothesis H2.1 and H3.1 is supported by the research.

**Table 7:** Regression analysis of the mediators.

Variable	Model 1: risk perception			Model 2: institution trust		
	B	*p*	*β*	B	*p*	*β*
Independent variable						
Information release quality	0.345^**^	0.000	0.323	0.501^**^	0.000	0.529
Communication variables						
Communication subjects						
Experts	0.096	0.142	0.045	0.058	0.222	0.030
Self-media	0.057	0.426	0.024	0.013	0.800	0.006
Government	−0.031	0.629	−0.015	−0.098*	0.035	−0.052
Official media	−0.025	0.713	−0.011	−0.015	0.766	−0.008
Family and friends	0.113	0.149	0.044	0.051	0.366	0.023
Information channels						
Mobile apps	−0.089	0.230	−0.040	0.227^**^	0.000	0.116
Television	−0.111	0.078	−0.055	−0.100^*^	0.029	−0.055
Radio	0.043	0.540	0.019	0.042	0.399	0.021
Internet portals	−0.150^*^	0.018	−0.073	0.049	0.283	0.027
Controls						
Risk preference	0.076^**^	0.000	0.186	0.029^**^	0.001	0.081
Others’ behaviors	0.225^**^	0.000	0.199	0.217^**^	0.000	0.217
Gender (male)	0.046	0.454	0.022	−0.150^**^	0.001	−0.082
Age	0.034	0.451	0.022	−0.004	0.897	−0.003
Education	−0.015	0.647	−0.013	−0.060*	0.010	−0.062
Disaster experience	0.052	0.529	0.018	0.024	0.695	0.009
Regional risk level	0.023	0.699	0.011	−0.025	0.564	−0.014
Regional fixed effects	Yes			Yes		
Constant	1.874**	0	—	0.748**	0	–
Adjust_*R*^2^	0.283			0.520		
*F*-value & *p*-value	*F* (31,894) = 12.755			*F* (31,894) = 33.343		
	*p* = 0.000			*p* = 0.000		

*p < 0.05, **p < 0.01. The mean VIF of all models is less than 2.

Stepwise regression was employed to examine the dependent variables (see Table 8). In the first step, the results from Model 3 (regression between independent and dependent variables) reveal that individuals perceiving higher quality of information release are more likely to engage in proactive protective behavior (*β* = 0.495**).

**Table 8:** Regression analysis of the dependent variable.

Variable	Model 3: protective behavior			Model 4: protective behavior		
	B	*p*	*β*	B	*p*	*β*
Independent variable						
Information release quality	0.443**	0.000	0.495	0.240**	0.000	0.268
Mediators						
Risk perception				0.128**	0.000	0.153
Institution trust				0.318**	0.000	0.336
Communication variables						
Communication subjects						
Experts	0.070	0.100	0.039	0.040	0.302	0.022
Self-media	−0.025	0.600	−0.012	−0.036	0.393	−0.018
Government	−0.034	0.416	−0.019	0.001	0.979	0.001
Official media	0.084	0.062	0.045	0.092*	0.023	0.049
Family and friends	0.095	0.064	0.044	0.064	0.164	0.030
Information channels						
Mobile apps	0.149**	0.002	0.080	0.088*	0.046	0.047
Television	−0.053	0.196	−0.031	−0.007	0.844	−0.004
Radio	−0.090*	0.047	−0.048	−0.109**	0.008	−0.058
Internet portals	−0.013	0.761	−0.007	−0.009	0.810	−0.005
Controls						
Risk preference	0.012	0.138	0.035	−0.007	0.341	−0.021
Others’ behaviors	0.288**	0.000	0.304	0.190**	0.000	0.201
Gender (male)	−0.115**	0.004	−0.067	−0.073*	0.043	−0.042
Age	0.011	0.699	0.009	0.008	0.753	0.006
Education	−0.018	0.396	−0.02	0.003	0.875	0.003
Disaster experience	−0.071	0.192	−0.03	−0.085	0.082	−0.036
Regional risk level	−0.003	0.936	−0.002	0.002	0.958	0.001
Regional fixed effects	Yes			Yes		
Constant	1.657**	0.000	—	0.862**	0.000	—
Adjust_*R*^2^	0.562			0.646		
*F*-value & *p*-value	F (31,894) = 39.356			*F* (33,892) = 52.044		
	*p* = 0.000			*p* = 0.000		

*p < 0.05, **p < 0.01. The mean VIF of all models is less than 2.

In the second step, the results from Model 4 (regression between independent, mediating, and dependent variables) show that both perceived risk (*β* = 0.153**) and institution trust (*β* = 0.336**) positively influence protective behaviors. After accounting for the mediating variables, the standardized coefficient for Information Release Quality is 0.268**.

In the third step, Bootstrap analysis was conducted to assess the mediating effect, utilizing a sample size of 5,000 with a 95% confidence interval. The results (see Table 9) indicate that the confidence interval for both risk perception and institution trust does not include 0 at the 95% confidence level, confirming a significant mediating effect and validating H2.3 and H3.3.

**Table 9:** Bootstrap test for the mediation effect.

Pathways	C	a	b	a*b	a*b (95% BootCI)	c′	Results (proportion of effect)
Information release quality ↓ Risk perception ↓ Protective behavior	0.443^**^	0.345^**^	0.128^**^	0.044	[0.028,0.071]	0.240^**^	H2.3 √(9.953%)
Information release quality ↓ Institution trust ↓ Protective behavior	0.443^**^	0.501^**^	0.318^**^	0.159	[0.134,0.224]	0.240^**^	H3.3 √(35.894%)

Finally, the regression results from Model 4 reveal that official media (*β* = 0.049*) and the use of mobile client apps (*β* = 0.047**) positively influence public protective behaviors. However, the influence of other authoritative sources, such as government and experts, was not significant.

**Figure 6:**
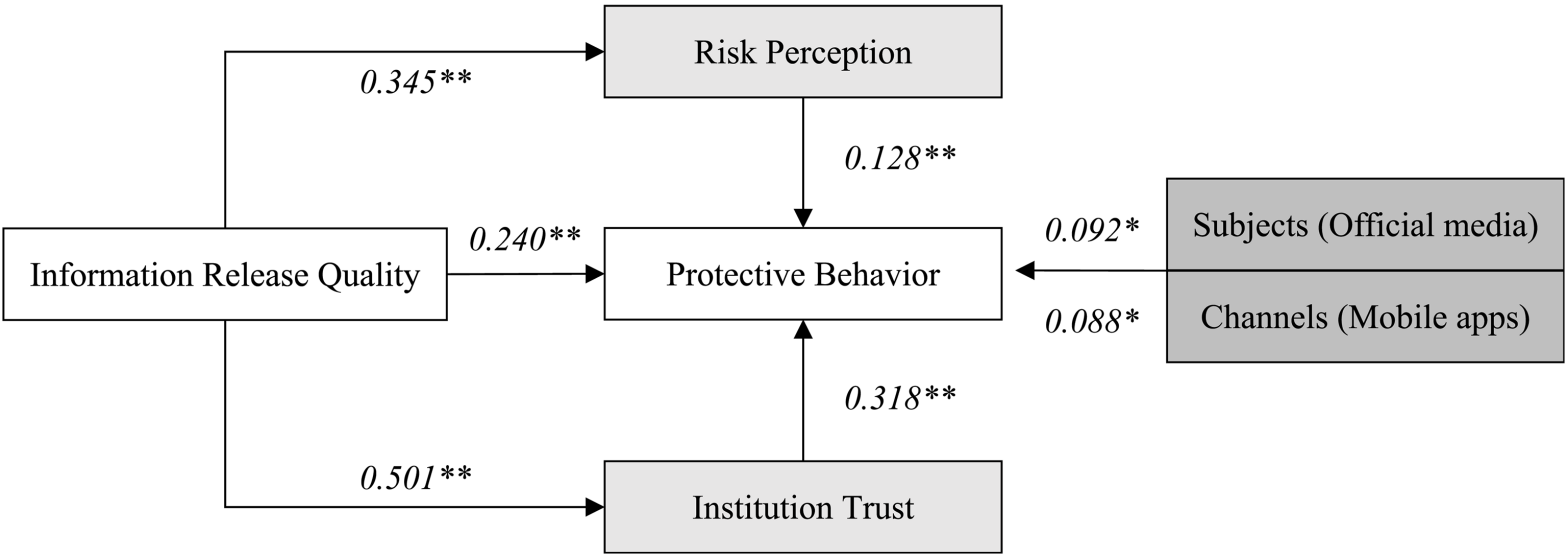
The research results.

The findings in Figure 6 highlight important factors influencing public protective behavior during epidemic responses. The mediation analysis result indicates the following finding.

1. Government information release and public protective behavior

Government communication has a significant impact on public protective behavior, primarily through two key pathways:

(1) Risk perception: when the government provides clear and reliable information about the epidemic, it influences how the public perceives the risks associated with the disease. Higher risk perception typically prompts individuals to adopt protective behaviors.
(2) Institution trust: in addition to risk perception, the study reveals that trust in institutions (i.e., the government) is a critical factor in motivating protective behaviors. People are more likely to follow health guidelines and engage in protective behaviors if they trust the institution disseminating the information.

(2) Impact of information channels and subject

Mobile Client Apps and Official Media were found to have a particularly strong influence on public protective behavior. These channels are effective in providing the public with timely, accessible, and credible information. Mobile applications (such as WeChat and Weibo) and official media help enhance public trust and ensure that messages about epidemic prevention are both seen and understood.

(3) Trust in Institutions vs. risk perception

While existing research often emphasizes the role of risk perception in shaping behavior, this study suggests that institution trust has a stronger impact in the Chinese context. Public trust in the government’s competence and transparency can lead to more proactive compliance with protective measures, even if individuals may not fully perceive the risk.

Therefore, this research underscores the critical role of government credibility in shaping public behavior. Strengthening trust in governmental institutions can enhance the public’s willingness to adopt recommended protective behaviors, ensuring a more effective response during epidemics. In conclusion, this research emphasizes that governments must prioritize both risk communication and the cultivation of institution trust to effectively guide public protective behaviors during public health crises.

## 6 Discussion and conclusions

Our research empirically demonstrates that during the normalization phase of COVID-19 in China, the quality of government information release significantly enhances public protective behaviors. This study has made three core innovations in theoretical perspective, mechanism exploration, and model construction. Firstly, in terms of research perspective, this study innovatively focuses on the quality of government information release (timeliness, responsiveness, and effectiveness), deepening the understanding of the core attributes of official risk communication. Secondly, in terms of theoretical mechanism, this study does not stay at the simple correlation between variables, but through rigorous empirical testing, clearly verifies the dual mediating path of “risk perception” and “institutional trust,” especially emphasizing that in the Chinese context, institutional trust is a more powerful driving factor. Thirdly, in terms of model construction, this study incorporates the communication process (information channels and sources) and social environment (other people’s behavior, regional risk) into the analysis framework, thus constructing an integrated model that integrates “information quality psychological cognition social context behavioral response”, filling the gap of insufficient consideration of complex social environment factors in existing research and enhancing the practical explanatory power of theoretical models.

The findings of this study engage in a constructive dialogue with recent advancements in risk communication and health behavior research. Current literature emphasizes that the public’s coping appraisal is more effective in promoting protective behaviors and reducing infections than solely emphasizing threats (86). The key mediating role of “institutional trust” identified in our study aligns closely with this perspective: high-quality official information, by building trust, essentially strengthens the public’s belief that “following recommendations is feasible and effective,” thereby reinforcing their coping appraisal. Second, recent research underscores the critical importance of looking beyond individual psychological factors to consider socio-structural contexts. Social Drivers constitute fundamental constraints on practicing protective behaviors (87). This supports the necessity of our study’s inclusion of social-contextual factors as moderators and suggests that future research should further explore how socioeconomic status interacts with information processing and trust to shape behavioral choices across different population groups. Furthermore, emerging studies reveal the contingent utility of psychological constructs such as risk perception and self-efficacy. Researchers apply the Health Belief Model and Protection Motivation Theory, respectively, to different populations, indicates that the predictive power of these constructs may vary across cultures, groups, or contexts (88, 93). This collectively underscores a central point: general behavioral models must be integrated with specific socio-psychological contexts, and the relative importance of their core pathways requires empirical examination. Finally, the importance of knowledge and targeted communication, implied in our focus on information quality, is underscored by research on other diseases. Alqaseer et al. (89) stress that nurses’ knowledge levels are crucial for effective management and public education, linking knowledge to demographic and professional factors. This parallels our argument that effective risk communication must be accurate and tailored, and suggests that training key intermediaries (like healthcare workers) is an essential component of the information dissemination ecosystem.

A number of recommendations are proposed. First, timely and Accurate Information Release. Governments must ensure the prompt release of accurate information regarding epidemics and corresponding control measures. It is vital to address public concerns, such as ensuring the availability of essential supplies and medical resources, alongside implementing effective disease control measures. This strategy helps the public assess risks objectively, reduce panic, counter misinformation, and maintain trust in the government, which fosters compliance with safety measures. Second, optimizing Risk Communication Methods. To strengthen the warning function of epidemic information, it is essential to optimize communication methods that highlight potential transmission risks. This allows the public to understand the current challenges and severity of the epidemic. Local governments should emphasize phrases like “Prevention and control efforts must remain vigilant” and “Individual responsibility for health is paramount,” utilizing clear language and prominent visuals, such as red imagery, to convey urgency. Additionally, communication guidelines should incorporate negative affect and risk indicators to signal heightened severity and future risks, especially when the likelihood of the epidemic spreading is high. Third, diversifying Communication Channels: Employing multiple channels to communicate policies from various perspectives improves public understanding and acceptance, encouraging the voluntary adoption of protective behaviors. Research indicates that mobile applications, such as Weibo and WeChat, are particularly effective channels for disseminating information and fostering public trust. Therefore, governments should leverage new media platforms strategically, ensuring frequent and timely updates with high-quality content. Successful operations of specific government accounts, like the Shenzhen Health Commission, demonstrate the effectiveness of these platforms. By offering real-time updates and clear communication, these platforms can elucidate policies, explain the scientific rationale behind them, and bridge the gap between the government and the public, enhancing policy authority and public cooperation.

This research has several limitations that should be acknowledged. First, while it focuses on the core constructs of government information quality, risk perception, and institutional trust, it does not fully explore a broader range of potential influences. Variables such as the stylistic and presentational aspects of information dissemination, broader social-environmental characteristics (e.g., community trust, cultural norms), and more granular individual traits (e.g., health literacy, specific personality factors) were not incorporated into the model. Second, the empirical analysis relies on cross-sectional survey data collected during a specific phase (the normalization period) of the COVID-19 pandemic in China. This design limits our ability to make strong causal inferences and to examine how the dynamics of public perception and behavior might evolve over different stages of a crisis. Third, the generalizability of the findings may be constrained by the specific socio-political and cultural context of China, where governmental authority and public communication channels have distinct characteristics.

To address these limitations and advance the current findings, future research could pursue several promising directions. First, studies should expand the explanatory framework by investigating the roles of unexamined variables, such as message framing, community-level social capital, and detailed individual differences, to provide a more holistic understanding. Second, employing longitudinal or experimental designs would be crucial to strengthen causal claims and to test the generalizability of the proposed model across different types of public health crises and cultural contexts. Third, integrating mixed-methods approaches would deepen the contextual analysis, using qualitative insights to explore how information is subjectively interpreted and how trust dynamically evolves, thereby enriching the quantitative model established here.

## Data Availability

The raw data supporting the conclusions of this article will be made available by the authors, without undue reservation.
